# Treatment of Facial Skin Laxity by a New Monopolar Radiofrequency Device

**DOI:** 10.4103/0974-2077.79178

**Published:** 2011

**Authors:** Uwe Wollina

**Affiliations:** *KHDF, Department Dermatology and Allergology, Dresden, Germany*

**Keywords:** Facial skin, radiofrequency, skin laxity

## Abstract

**Background:**

Acquired facial skin laxity seems to be a result of the combination of intrinsic and extrinsic processes. For treatment of facial ageing, non-invasive procedures have become popular.

**Aim:**

We wanted to investigate the effect of a new 2.2-MHz radiofrequency (RF) device on acquired facial skin laxity.

**Setting:**

Outpatient clinic associated with an academic teaching hospital.

**Materials and Methods:**

We performed an open trial with the RF-ReFacing™ device (Meyer-Haake Medical Innovations, Wehrheim/ Germany) in the monopolar mode with a power of 8-12 W, two passes per session and repetition three times after 2 weeks without treatment.

**Results:**

A total of 20 Caucasian female patients were included (age range, 34-73 years). The procedure was performed without any analgesia. We did not see any adverse effect. The procedure was scored as most convenient or convenient by all patients. Improvement in skin laxity and fine wrinkles was seen after the second treatment in 19 of the 20 patients and after the third treatment in 100% of the patients. On a scale from 0 to 3, improvement in the lower lid, Crow’s feet and jowl line was scored 2.6±0.7 by the patients; improvement in the overall appearance of the face was scored 2.3±0.5. Blinded assessment of the photographs rated the improvement as good or better in 15 of the 20 patients, moderate in 3 patients, no change in 1 patient.

**Conclusions:**

RF-ReFacing™ treatment was effective in improvement in skin laxity. Patients’ satisfaction was high. Although RF-ReFacing™ treatment cannot substitute surgical procedure, it might prolong the time to the first surgical facial lift. The number of patients treated was small, and no quantitative measurements or histopathology was performed. Hence further studies with greater number of patients are necessary.

## INTRODUCTION

Volume loss, skin laxity and wrinkling are major findings associated with facial ageing. Skin laxity can also be caused by several heritable connective tissue disorders. In the ageing skin, acquired skin laxity seems to be a result of the combination of intrinsic and extrinsic processes. A major extrinsic factor is ultraviolet radiation (UVR), which can be potentiated by smoking.[1]

For treatment of facial ageing, more and more minor invasive or non-invasive procedures have become popular, like fillers for volume enhancement and sculpturing, Fraxel ^TM^ laser, intense pulsed light or chemical denervation by botulinum toxins for wrinkles, and subdermal laser lipolysis for skin tightening.[2,3]

In recent years, radiofrequency has become another tool for facial rejuvenation. Radiofrequency current is formed when charged particles flow through a closed circuit. As the energy meets resistance in the tissue, heat is produced. The amount of heat will vary depending on the amount of current, the resistance levels in the targeted tissue and the characteristics of the electrodes. The amount of RF energy applied can be configured to target specific tissues. In addition, the water content of skin varies between different areas of the body, with time of the day, environmental humidity, internal hydration and the topical moisturizing agents used. Thus the flow of RF through the skin depends on multiple factors. High-impedance tissues, such as subcutaneous fat, generate greater heat and account for the deeper thermal effects of RF devices.[4]

It is estimated that adult skin loses approximately 1% of its dermal collagen content on an annual basis due to increased collagen degradation and decreased collagen synthesis. When the collagen fibres are heated, some of the cross-links are broken, causing the triple helix structure to unwind. Beyond a certain level, depending on a combination of maximal temperature and exposure time, collagen fibres undergo denaturation. When the cross-links are maintained, at least partially, collagen shrinkage and thickening are achieved.[5,6]

Based on this principle, treatments are designed to cause the shrinkage of dermal collagen using heat generated by a radiofrequency current. In addition, the treatment promotes the formation of new collagen via the natural wound-healing response of the skin and a direct effect on the dermal cellular matrix. The extent of collagen shrinkage, fibroblast activation, fibroplasia and overall collagenesis in the different skin layers is based on a complex multivariate mechanism, which depends on the temperature distribution and timing. This enables shrinkage at a certain depth, followed by collagenesis at a different, preferably more superficial layer. Exposure of the skin to heat is also known to increase blood perfusion in the affected area, supporting the fibroblast activity and the overall rejuvenation process.[5–7]

Transmission electron microscopy studies have shown immediate results of heating with regard to collagen denaturation, with a resultant fibril contraction and tissue thickening.[6] An inflammatory wound-healing response ensues with long-term neo-collagenesis, effecting rhytide reduction and further tissue contraction. In addition, selective heating and tightening of fibrous septae within the subcutaneous layer likely accounts for immediate contour changes in the skin after treatment.[8]

## MATERIALS AND METHODS

This was an open trial for facial laxity. Female patients older than 18 years of age, non-pregnant, non-lactating were included after obtaining informed consent. Patients with implanted pacemakers or defibrillator were not included, since this is an absolute contraindication for RF therapy. Patients with presence of acute systemic infections and local infections such as herpes simplex or impetigo and those with open wounds in the area of treatment were excluded. Patients with genetic disorders of connective tissue, like cutis laxa, were excluded from this trial.

We used the RF-ReFacing™ device based on the radioSURG™ equipment (Meyer-Haake Medical Innovations, Wehrheim/ Germany) in the monopolar mode. This equipment generated a frequency of 2.2 MHz. Monopolar systems deliver current through a single contact point with an accompanying grounding pad that serves as a low resistance path for current flow to complete the electrical circuit.

Patients were treated in a lying position with the large neutral electrode placed beneath the shoulder. In most cases, a large ball electrode (diameter, 8 mm) was employed for facial treatment. In some cases, a cone-shaped electrode (diameter, 15 mm; cone height, 20 mm) was used.

The skin was covered by a facial moisturizing cream, and the facial parts were treated by two passes per session with an interval of 2 weeks for a total of three sessions. The power was adjusted to the patient’s comfort to get the sense of a light, convenient warming of tissue, e.g., 8-12 W. In this setting, no cooling of skin was necessary. Photos were taken before and after each session. Patients were asked about their satisfaction. A blinded score of the photos was performed by a doctor not involved in treatment. Scoring was carried out in a 3-step mode: 0 - no change; 1 - moderate improvement; 3 - marked improvement.

## RESULTS

A total of 20 female patients were included (age range, 34-73 years; mean age, 41±11 years). Four patients were smokers; none was attending a tanning saloon. All patients were Caucasians with photo-skin type I to III; the majority (*n*=13) had a skin type II.

The procedure did not need any analgesia or anaesthetics. We did not see any adverse effect, such as burning, ecchymosis or pigmentary changes. Treatment took only 10 minutes per session. The procedure was scored as most convenient or convenient by all patients. There was no down-time for patients.

**Figure 1:**
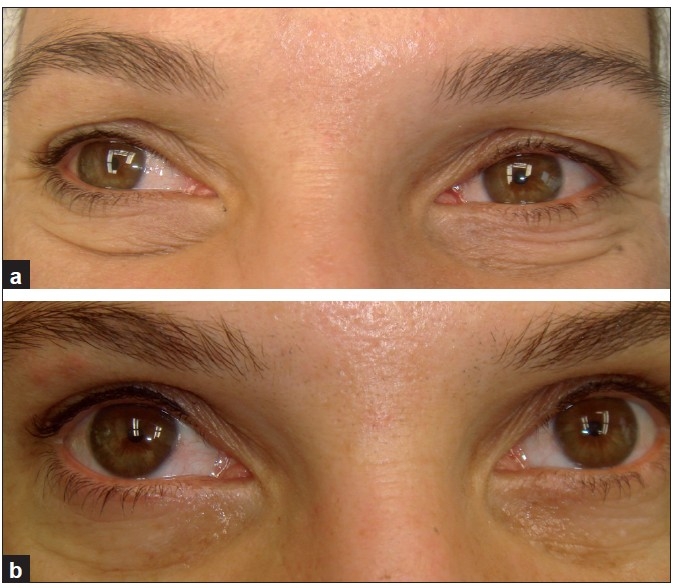
A 36-year-old woman. (a) Before treatment; and (b) after three treatments, with smoothing and tightening of lower lids

**Figure 2:**
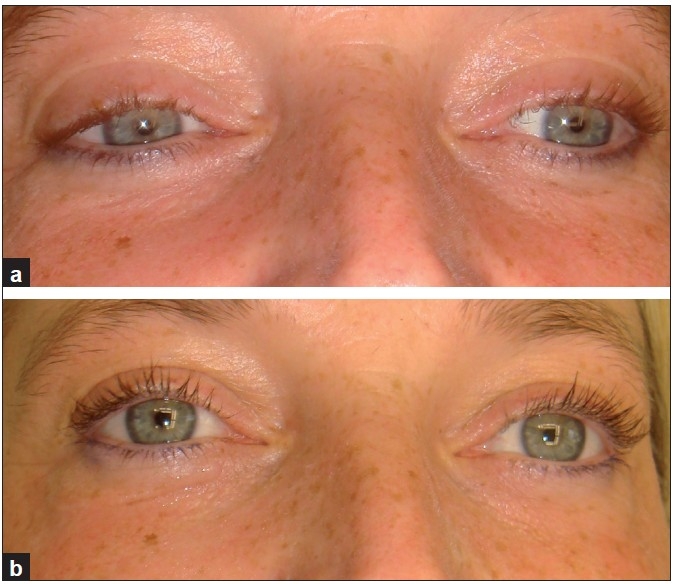
A 45-year-old woman. (a) Before treatment; and (b) after second treatment, with improvement in lower lids

**Figure 3:**
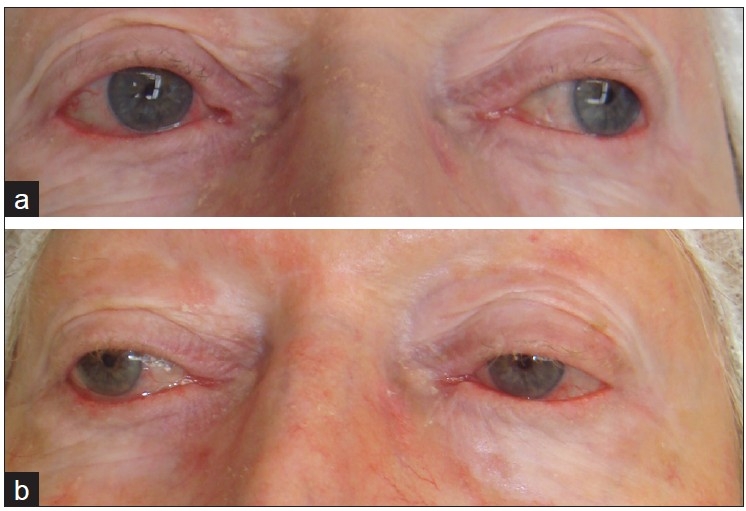
A 73-year-old woman. (a) Before treatment; and (b) after second treatment, with mild improvement in lower lids

**Figure 4:**
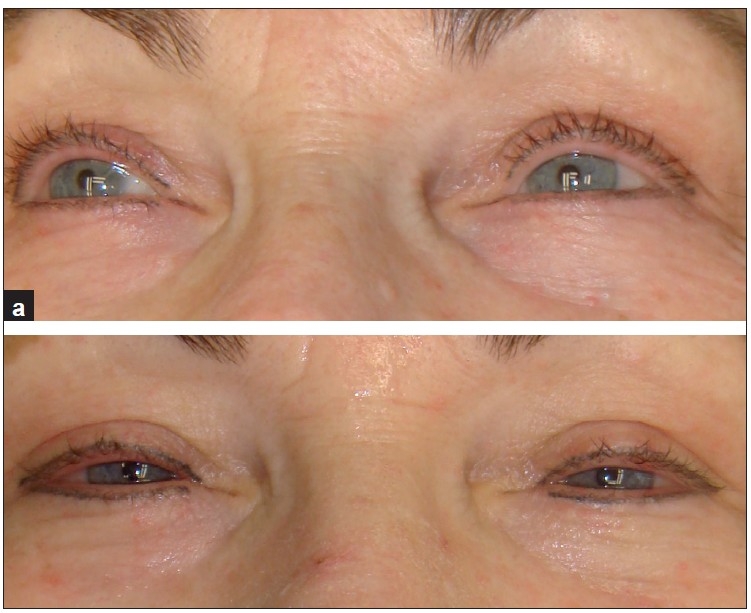
A 67-year-old woman. (a) Before treatment; and (b) after second treatment, with improvement in tear-trough

Improvement in skin laxity and fine wrinkles was seen after the second treatment in 19 of the 20 patients and after the third treatment in 100% of the patients. On a scale from 0 to 3 (worsening, no change, mild change, moderate-to-marked change, respectively), improvement in the lower lid, Crow’s feet and jowl line was scored 2.6±0.7 (average score) by patients. Improvement in the overall appearance of the face was scored 2.3±0.5. The blinded assessment of the photographs rated the improvement as good or better in 15 of the 20 patients, moderate in 3 patients, no change in 1 patient. Results are shown in Figures 1–3 for lower lids; and in Figure 4, for tear-trough. Although the clinical effect is age dependent, improvement could be seen also in patients older than 60 years of age. The strongest effects were achieved for lower lids (improvement score, 2.8±0.6), followed by Crow’s feet (2.7±0.6) and jowl line (2.4±0.7). The improvement in Crow`s feet and lower eye lids reached significance comparing the situation before and after the third treatment (*P*=0.04; one-sided Student’s *t*-test) but not for the jowl line (*P*=0.056).

**Figure 5:**
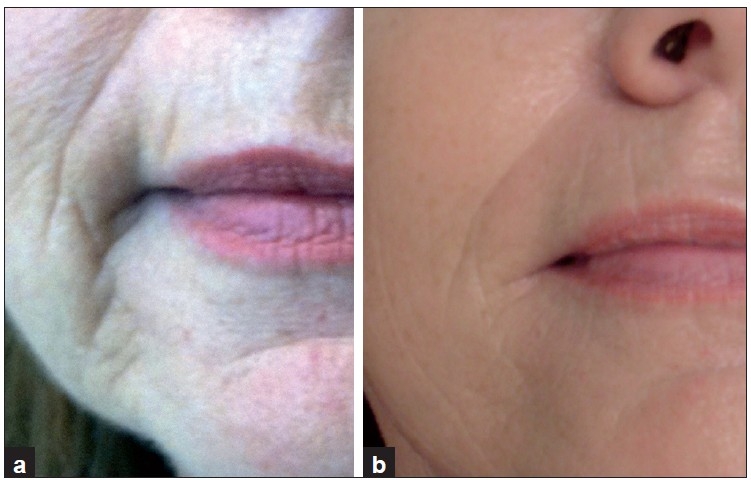
A 59-year-old woman, perioral lines. (a) Before treatment; (b) after third treatment, with improvement in the lines

**Figure 6:**
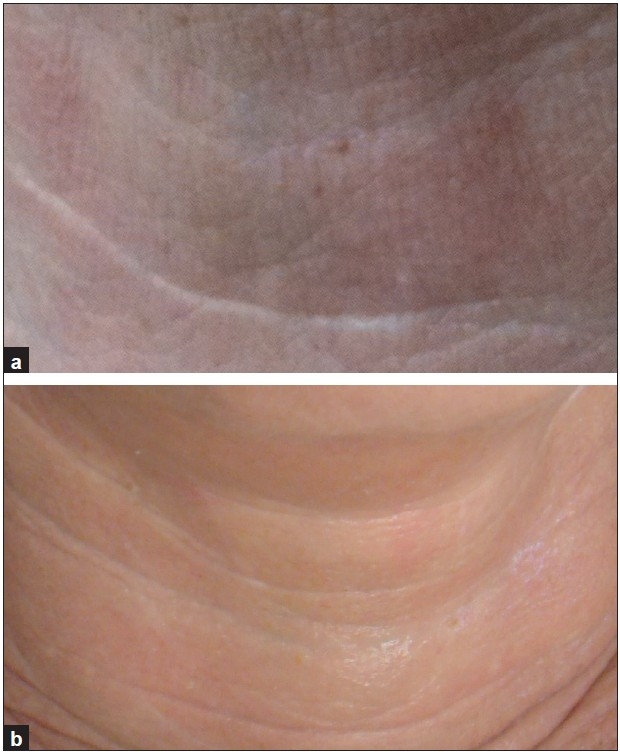
A 59-year-old woman, neck. (a) Before treatment; (b) after third treatment, with improvement in neck lines

In addition to the periocular skin, we noted improvement in cheeks, lower face and neck in several patients as well [Figures 5 and 6].

Effects were obvious but not dramatic. Nevertheless, patients’ satisfaction was high. The RF-ReFacing™ treatment may be used in combination with other procedures for better results but was used only as a monotherapy in this study.

## DISCUSSION

In a monopolar configuration, one electrode is active and the other (a considerably larger one) is placed far from the first one and serves as a grounding pad. The main advantage of monopolar delivery is the concentration of a high-power density on the surface of the electrode and the relatively deep penetration of the emitted power. In contrast to laser, monopolar RF offers volumetric heating. The depth of heating is dependent upon the size and geometry of the treatment tip being used. A conductive coupling fluid is used during the treatment to enhance the thermal and electrical contact between the treatment tip and the skin. Monopolar electrodes concentrate most of their energy near the point of contact, and energy rapidly diminishes as the current flows toward the grounding electrode. Using electrode configurations like large-ball or cone electrodes, and adjust the current penetration can be balanced according to the needs of facial skin. A facial moisturizing cream was used for conductive coupling.

RF devices have shown clinical utility in aesthetic medicine for the treatment of excessive facial laxity and rhytide reduction. In particular, these systems have proven effective for the reduction of brow ptosis, prominent mentolabial folds and cheek laxity.[9–16] Since the heating is not chromophore dependent, the procedure is also safe for ethnic skin.[17]

The present trial included Caucasian patients with Fitzpatrick skin types I-III. We observed a marked improvement in skin laxity and fine wrinkling, in particular on the lower eye lids and the Crow’s feet. Adverse effects were not noted. The treatment is safe and convenient. It is not time consuming and may be performed as a lunch procedure. Our results are supported by a smaller study by Ruiz-Esparza on non-invasive lower eye lid blepharoplasty by non-ablative radiofrequency in 9 patients.[18] In contrast to his procedure, in this trial no analgesia was used.

An option for further improvement of outcome might be the multi-pass vector RF therapy as suggested by Finzi and Spangler.[19] By this technique, more oedema was noted, but severe adverse effects were not seen. In this trial, we limited the application to two passes only. More passes might increase efficacy, but this has not been investigated in detail.

Another major advantage of monopolar RF is its applicability to various age groups. Although the final outcome might be better in biologically younger facial skin, there is no age limit. Nevertheless, when a surgical intervention is necessary, RF is not a substitute. On the other hand, RF technique might prolong the time to the first surgical facial lift.

This study has some limits — small number of patients, broad age range, no objective measurements of laxity. Therefore, multi-centre trials would be most useful to increase the number of patients and introduce more sophisticated measurements of the effect. Within these limits, the monopolar RF device seems to be a useful tool with low costs and high levels of patient satisfaction.

## References

[1] Wasserman D, Avram MA, Katz BE, Sadick NS, Dover JS, Alam M (2010). Pathophysiology of skin laxity and cellulite. Body Contouring. Procedures in Cosmetic Dermatology.

[2] Goldman A, Shavelzon D, Blugerman G (2002). Laser lipolysis: Liposuction using Nd:YAG laser. Rev Soc Brasil Chir Plast.

[3] Goldman A (2006). Submental Nd:YAG laser-assisted liposuction. Lasers Surg Med.

[4] Arnoczkay SP, Aksan A (2000). Thermal modification of connective tissues: Basic science considerations and clinical implications. J Am Acad Orthop Surg.

[5] Sadick NS, Makino Y (2004). Selective electro-thermolysis in aesthetic medicine: A review. Lasers Surg Med.

[6] Zelickson BD, Kist D, Bernstein E, Brown DB, Ksenzenko S, Burns J (2004). Histological and ultrastructural evaluation of the effects of a radiofrequency-based nonablative dermal remodeling device: A pilot study. Arch Dermatol.

[7] Dierickx CC (2006). The role of deep heating for noninvasive skin rejuvenation. Laser Surg Med.

[8] Homoth J, Wenderoth M, Druga T, Winking L, Ulbrich RG, Bobish CA (2009). Electronic transport on the nanoscale: Ballistic transmission and Ohm’s law. Nano Lett.

[9] Hsu TS, Kaminer MS (2003). The use of nonablative radiofrequency technology to tighten the lower face and neck. Semin Cutan Med Surg.

[10] Fitzpatrick R, Geronemus R, Goldberg D, Kaminer M, Kilmer S, Ruiz-Esparza J (2003). Multicenter study of noninvasive radiofrequency for periorbital tissue tightening. Lasers Surg Med.

[11] Alster TS, Tanzi E (2004). Improvement of neck and cheek laxity with a nonablative radiofrequency device: A lifting experience. Dermatol Surg.

[12] Nahm WK, Su TT, Rotunda AM, Moy RL (2004). Objective changes in brow position, superior palpebral crease, peak angle of the eyebrow and jowl surface area after volumetric radiofrequency treatments to half of the face. Dermatol Surg.

[13] Fritz M, Counters JT, Zelickson BD (2004). Radiofrequency treatment for middle and lower face laxity. Arch Facial Plast Surg.

[14] Koch RJ (2004). Radiofrequency nonablative tissue tightening. Facial Plast Surg Clin North Am.

[15] Weiss RA, Weiss MA, Munavalli G, Beasley KL (2006). Monopolar radiofrequency facial tightening: A retrospective analysis of efficacy and safety in over 600 treatments. J Drugs Dermatol.

[16] Alexiades-Armenakas M, Rosenberg D, Renton B, Dover J, Arndt L (2010). Blinded, randomized, quantitative grading comparison of minimally invasive, fractional readiorequency and surgical face-lift to treat skin laxity. Arch Dermatol.

[17] Kushikata N, Negishi K, Tezuka Y, Takeuchi K, Wakamatsu S (2005). Non-ablative skin tightening with radiofrequency in Asian skin. Lasers Surg Med.

[18] Ruiz-Esparza J (2004). Noninvasive lower eyelid blepharoplasty: A new technique using nonablative radiofrequency on periorbital skin. Dermatol Surg.

[19] Finzi E, Spangler A (2005). Multipass vector (mpave) technique with nonablative radiofrequency to treat facial and neck laxity. Dermatol Surg.

